# Cardiometabolic risk factors and their association with carotid intima media thickness among patients with type 2 diabetes mellitus

**DOI:** 10.55730/1300-0144.6030

**Published:** 2025-04-07

**Authors:** Christian SALEH

**Affiliations:** Neurologist, independent researcher, Basel, Switzerland

**Keywords:** Ultrasound, carotid artery, surrogate marker, carotid intima-media thickness

## Abstract

Comment to Weerarathna TP, Lekamwasam S, Kodikara I, Wasana KGP, Fonseka L. Control of cardiometabolic risk factors and their association with carotid intima media thickness among patients with type 2 diabetes mellitus-single center experience in a developing country. (Turk J Med Sci. 2024 Jan 11; 54 (3): 545–554)

In a recent issue, Weerarathna et al. published a study titled “Control of cardiometabolic risk factors and their association with carotid intima media thickness among patients with type 2 diabetes mellitus-single center experience in a developing country” [1]. The aim of the study was to investigate the degree of atherosclerotic cardiovascular disease (ASCVD) risk factor control and its association with carotid intima-media thickness (CIMT) in patients with type 2 diabetes mellitus [1]. CIMT was used as a sonographic surrogate marker of preclinical atherosclerosis. The study enrolled 300 patients (209 women and 91 men), with a mean age of 62 years (SD: 10) [1]. CIMT was measured at the far wall of the common carotid artery (CCA) [1]. The study found statistically significant differences in CIMT between optimally controlled triglyceride (TG) and suboptimally controlled TG group (p = 0.027), as well as between the groups with and without nonalcoholic fatty liver disease (NAFLD) (p = 0.045), after adjustment for age and duration of diabetes [1]. The authors concluded, “This study indicates that LDL-C, TG, and the presence of NAFLD are significant predictors of chronic arterial injury in patients with T2DM” [1]. Several critical details regarding the CIMT measurement methodology are missing, preventing a balanced evaluation of the study’s results.

## 1. Cardiac synchronization

Importantly, CIMT measurements need to be synchronized with the cardiac cycle and performed during the end-diastolic phase [2,3]. Due to vessel diameter variations, consequent to different cardiac phases, mean differences of up to 0.041 mm between systolic and diastolic phases have been reported [3]. However, Weerarathna et al. [1] did not address this critical methodological aspect.

## 2. Manual versus semiautomated based measurement

The measurements in Weerarathna et al.’s study [1] appear to have been performed manually, rather than using semiautomated methods, thereby introducing an additional source of inaccuracy [4]. Furthermore, Weerarathna et al. [1] did not report on intra- and interoperator variability [5,6].

## 3. Measurement protocols

CIMT measurement protocols vary between centers: one recommendation is to measure at the far wall of the CCA, near the CA bifurcation [2]. It should be noted that the recommendation to measure CIMT at the far wall is primarily based on technical reasons, particularly the higher spatial resolution it provides [2]. However, atherosclerosis presents asymmetrically [7], and measuring a single predetermined CA segment may coincide with a normal area, overlooking segments with atherosclerotic changes. Therefore, measuring CIMT at multiple sites, including both arterial walls and various CA segments (e.g., CCA, bifurcation, and internal CA), may yield a more accurate assessment [8–10].

In summary, minor inaccuracies caused by suboptimal measurement protocols or techniques can quickly accumulate, resulting in an inaccurate CIMT estimate (with a normal reference range of 0.6 to 0.9 mm). More importantly, such submillimeter inaccuracies can be consequently sufficient to misclassify patients into different CIMT categories. A meticulous and detailed measurement protocol needs to be in place when using CIMT. In light of these considerations, the CIMT data and related conclusions of Weerarathna et al.’s study [1] should be analyzed with caution.
